# Effects of genotype and age on eggshell cuticle coverage and color profile in modern laying hen strains

**DOI:** 10.1016/j.psj.2021.101691

**Published:** 2021-12-30

**Authors:** F. Sirri, M. Zampiga, A. Berardinelli

**Affiliations:** ⁎ Department of Agricultural and Food Sciences, Alma Mater Studiorum - University of Bologna, 40064 Ozzano dell'Emilia, Italy; † Department of Industrial Engineering, University of Trento, 38123 Povo, TN, Italy; ‡ Centre Agriculture Food Environment, University of Trento, 38010 S. Michele all'Adige, TN, Italy

**Keywords:** hen genotype, age, eggshell cuticle, color, egg

## Abstract

The aim of this research was to investigate the effects of laying hen genotype and age on eggshell cuticle deposition. A total of 4,320 brown eggs were obtained from 3 modern hen strains (A, B, and C), currently used worldwide for commercial egg production, at different intervals of age (20–30, 40–50, and 60–70 wk). Four samplings of 120 randomly collected eggs were carried out for each genotype/interval of age. Eggs were individually weighed and cuticle blue staining was used to assess quality and degree of cuticle coverage. On each egg, the eggshell color profile was assessed before and after staining using the CIE L*a*b* system and these values were used to calculate ΔE*_ab_. A 4-point scale visual score (**VS**) system was also applied to estimate the degree of cuticle coverage after staining (0 = no coverage, 1 = partial coverage, 2 = total coverage - low degree, 3 = total coverage - high degree). The effects of genotype and age and their interaction on eggshell color attributes were assessed by means of factorial ANOVA, while omnibus Chi-Square and Chi-squared Automatic Interaction Detector algorithm were applied for the analysis of VS data. Overall, both genotype and age affected the eggshell color profile as well as the degree of cuticle coverage. Hen strain A showed better cuticle deposition in comparison with B and particularly C one, being ΔE*_ab_ values significantly higher. The VS evaluation revealed that eggs with impaired cuticle coverage degree increased with the hen age (23, 34, and 37%, respectively for 20–30, 40–50, and 60–70 wk; *P* < 0.05). However, a significant interaction between genotype and age was observed: transition from early to late hen age resulted in a significantly different pattern of ΔE*_ab_ changes in each genotype. The classification tree analysis confirmed that the hen genotype has a greater effect than the age on cuticle deposition. In conclusion, considering the importance of the cuticle in table egg production, these results highlight the crucial role exerted by the genotype on eggshell cuticle coverage.

## INTRODUCTION

The eggshell cuticle is the outer layer deposited on the eggshell during the final stages of egg formation (1–1.5 h before deposition) (Baker and Balch, 1962; Sparks and Board, 1985). It presents a variable thickness (about 12 µm) and it is composed by 85 to 90% of proteins (mainly glycoproteins), polysaccharides (4%), and lipids (3%) (Nys et al., 1999; Rose-Martel et al., 2012; Rodríguez-Navarro et al., 2013). Beside its role as modulator for the gaseous exchanges, the cuticle represents the first line of defense against microbial penetration into the egg, thus limiting potential contamination of the inner egg contents by *Salmonella* spp., *Bacillus cereus* and *Escherichia coli* (Sparks, 1994; De Reu et al., 2006; Scallan et al., 2011; Bain et al., 2013; Gole et al., 2014; Muñoz et al., 2015; Bain et al., 2019; Chen et al., 2019). It has been demonstrated that eggs with poor cuticle were more often penetrated by a laboratory strain of *Escherichia coli* than eggs with good cuticle as well as that cuticle score is a significant predictor of Salmonella eggshell penetration (Bain et al., 2013; Gole et al., 2014).

In many countries, including United States, Australia, Japan, and Sweden, egg washing with chemicals such as sodium carbonate and sodium hypochlorite is an established practice to sanitize table eggs (Leleu et al., 2011; Kulshreshtha et al., 2018), which has proven to be effective in reducing Enterobacteriaceae count on the eggshell (May et al., 2013). However, egg washing can damage or partially remove the cuticle thereby increasing the risk of bacteria penetration into the egg, and can hidden poor husbandry practices and hygiene standards (European Food Safety Authority, 2005; Kulshreshtha et al., 2018). For such reasons, no disinfection treatments are currently allowed in Europe according to the legislation in force (Commission Regulation, 2008), which states that ‘…class A eggs should not be washed because of the potential damage to the physical barriers, such as the cuticle, which can occur during or after washing…’. In the EU scenario, but also in other countries where egg washing is not allowed, the issue of cuticle quality is of vital importance as the safety of table eggs relies to a large extent on the natural defense systems of the egg, which include the cuticle.

In general, the cuticle quality and the degree of coverage are affected by many factors including the hen age and its genetic background, as well as the husbandry system and the egg storage conditions (Hannah et al., 2011; Bain et al., 2013, 2019; Rodríguez-Navarro et al., 2013; Dunn et al., 2019). The potential genetic contribution to eggshell cuticle coverage in commercial poultry species has been highlighted in different studies. Bain et al. (2013, 2019) demonstrated that the quantity of cuticle is a heritable trait in chickens. A meta-analysis study indicated that the estimated heritability of cuticle deposition was moderately high (0.38) both in pure breeds (Rhode Island Red, 2 White Leghorns, White Rock) and in a commercial broiler line (Dunn et al., 2019). On the other hand, Bain et al. (2013) also reported that the use of artificial incubation in commercial practices could have reduced the selection pressure for cuticle quality in modern chicken genotypes, with potential consequences on product quality and safety as well as risk of pathogen outbreaks. However, the information regarding the effect of the genotype on cuticle deposition capacity and its persistence over the laying period is still sparse, particularly for modern hen strains. To address these concerns, the present study was aimed at evaluating the quality and degree of cuticle deposition as well as the eggshell color profile in 3 modern hen strains, which are currently used worldwide for commercial egg production, in representative intervals of their laying cycle.

## MATERIALS AND METHODS

### Eggs Sampling and Analysis

**Figure 1:**
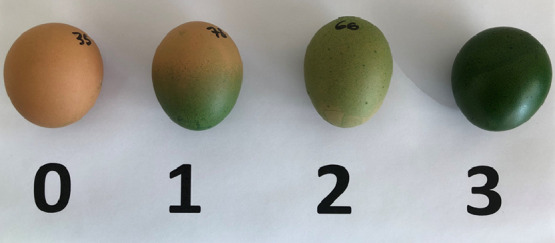
Criteria adopted for the visual score (VS) evaluation based on a 4 point-scale (0 = no cuticle coverage; 1 = partial cuticle coverage; 2 = total cuticle coverage – low degree; 3 = total cuticle coverage – high degree).

A total of 4,320 brown eggs obtained by three modern laying hen strains (A, B, and C), currently used for commercial egg production and distributed worldwide, were collected at 3 different intervals of hen age (20–30, 40–50, and 60–70 wk) in Italian commercial farms located in a similar geographical area and controlled by a large integrator group. The three genotypes were not genetically related and received the same commercial diet according to the feeding program of the company (Table 1). For each interval of age, 4 samplings of 120 randomly collected eggs were carried out for each genotype both from cage and barn systems (50% cage - 50% barn; Table 2). Eggs were collected at the day of deposition and then transferred to the lab where they were stored at room temperature. The following day, eggs were singularly weighed and eggshell color “before staining” was assessed in 2 different positions (i.e., above and below the equator region) using a reflectance colorimeter equipped with illuminant source C (Minolta CR-300, Minolta Italia S.p.A., Milano, Italy). The results were expressed according to the CIE L*a*b* system (CIE, 1978) where L* stands for lightness, a* for redness and b* for yellowness. To assess the quality and degree of eggshell cuticle coverage, eggs were stained by immersion into a solution of 1% of Cuticle Blue staining (MS Technologies Ltd., Northamptonshire, UK) for 5 min, rinsed in clean water to remove the excess of stain, and dried for 24 h at room temperature. Then, the eggshell color profile “after-staining” was assessed in the same 2 positions as described above. The average values obtained before and after staining were used to calculated ΔE*_ab_ according to the following formula (CIE, 1978):

$$\Delta E\begin{array}{c} {}*\\ ab\; \end{array}=\;\sqrt{{\left(L\begin{array}{c} {}*\\ 2 \end{array}-L\begin{array}{c} {}*\\ 1 \end{array}\right)}^{2}+{\left(a\begin{array}{c} {}*\\ 2 \end{array}-a\begin{array}{c} {}*\\ 1 \end{array}\right)}^{2}+{\left(b\begin{array}{c} {}*\\ 2 \end{array}-b\begin{array}{c} {}*\\ 1 \end{array}\right)}^{2}\;}$$

where “2” and “1” refer respectively to the colorimetric assessments conducted after and before staining. In brief, the higher the ΔE*_ab_ values, the better the staining affinity and thus the higher the eggshell cuticle quality. In addition, a 4-point scale visual score (**VS**) system was developed to estimate the degree of cuticle coverage after staining according to the following criteria: 0 = no cuticle coverage, 1 = partial cuticle coverage, 2 = total cuticle coverage, 3 = very uniform total cuticle coverage (Figure 1). In order to be more explicative, scores 0 and 1 (no and partial cuticle coverage, respectively), as well scores 2 and 3 (total cuticle coverage - low degree and total cuticle coverage - high degree, respectively), were merged and data analyzed as described below.

**Table 1:** Composition of the commercial diets according to different feeding phases.

Ingredients, %	20–30 wk	40–50 wk	60–70 wk
Corn	52.8	55.3	58.2
Soybean meal	27.8	25.6	23.1
Full-fat soybean	5.0	5.0	5.0
Soybean oil	1.8	1.3	0.8
Calcium carbonate	9.9	10.3	10.8
Dicalcium phosphate	1.5	1.3	1.0
Sodium bicarbonate	0.30	0.30	0.30
Sodium chloride	0.18	0.18	0.15
L-lysine	0.12	0.11	0.10
DL-Methionine	0.25	0.20	0.15
Phytase	0.06	0.06	0.06
Vitamin and mineral premix^1^	0.30	0.30	0.30
Calculated analysis			
Dry matter (%)	89.0	89.0	89.0
Crude protein (%)	19.2	18.1	17.2
Total fat (%)	4.7	4.4	4.2
Crude fiber (%)	3.5	3.5	3.5
Ash (%)	12.8	13.0	13.5
Calcium (%)	4.0	4.3	4.5
Phosphorus (%)	0.65	0.60	0.55
ME (kcal/kg)	2.720	2.700	2.680

1 Provided the following per kg of diet: vitamin A (retinyl acetate), 11,000 IU; cholecalciferol, 3,000 IU; DL-α_tocopheryl acetate, 40 IU; menadione sodium bisulfite, 3.3 mg; riboflavin, 6.0 mg; pantothenic acid, 11.0 mg; niacin, 30 mg; pyridoxine, 4 mg; folic acid, 1 mg; biotin, 0.05 mg; thiamine, 2.5 mg; vitamin B12 20 μg; Mn, 15 mg; Zn, 50 mg; Fe, 30 mg; Cu, 6 mg; I, 1.5 mg; Se, 0.2 mg; ethoxyquin, 100 mg.

**Table 2:** Egg sampling scheme.

Hen genotype	Hen age (wks)	Sampling (n.)	Eggs/sampling (n.)	Total eggs analyzed (n.)
A	20–30	4	120	480
	40–50	4	120	480
	60–70	4	120	480
B	20–30	4	120	480
	40–50	4	120	480
	60–70	4	120	480
C	20–30	4	120	480
	40–50	4	120	480
	60–70	4	120	480

### Statistical Analysis of Data

The data analysis was conducted by using the software package SPSS 20.0 (IBM, NY). The effects of genotype and age and their interaction on measured and calculated eggs attributes were explored by using Factorial Analysis of Variance (ANOVA) (*P* < 0.05). Multiple comparisons among intervals of ages (20–30, 40–50, and 60–70 wk; within the same genotype) and among genotypes (A, B and C; within the same interval of age) mean values were conducted according to Tukey HSD test (*P* < 0.05). Spearman's correlation coefficient (ρ) was calculated and discussed for bivariate correlation analysis between VS and ΔE*_ab_ colorimetric attribute. The omnibus Chi-Square statistics was used to test the effects of the independent categorical variables “genotype” and “interval of age” on the VS variable. A post hoc analysis using standardized residuals and the percentage of relative and absolute contributions of the individual cells was also conducted. The standardized residual for each cell was calculated and discussed to evidence which discrepancies between observed and expected values are larger than those might be expected by chance. The relative contribution method was computed by dividing each cell chi-square by the omnibus chi-square value, while for the absolute contribution each cell chi-square was divided by the numerosity of the sample (Beasley and Schumacker, 1995). The relationship between the categorical variables “genotype” and “interval of age” (explanatory variables) and the occurrence of the VS (response variable) was assessed using a tree-based model, the Chi-squared Automatic Interaction Detector (**CHAID**) algorithm (Kass, 1980). In CHAID, the explanatory variable was split into subgroups according to the response variable. The variable with the highest chi-square value was selected and each subgroup was independently analyzed to produce further subgroups. The data model proceeded stepwise and used *P*-values with a Bonferroni correction as splitting criteria.

## RESULTS AND DISCUSSION

Mean values of egg quality attributes before and after cuticle staining according to hen genotype and interval of age are summarized in Tables 3 and 4, respectively. A significant effect of both variables “genotype” and “interval of age” and their interaction was observed for all measured attributes.

**Table 3:** Effect of hen genotype (A, B and C) on egg weight, eggshell colorimetric attributes before cuticle staining, and ΔE*_ab_ values at 20–30, 40–50, and 50–70 wks of hen age.

Age, wks	Genotype	Egg weight, g		Lightness - L*		Redness - a*		Yellowness - b*		ΔE*_ab_	
20–30	A	60.5 (4.8)	^AB^	57.6 (3.3)	^C^	18.4 (2.1)	^A^	30.6 (1.9)		26.1 (6.8)	^A^
	B	61.4 (4.5)	^A^	63.3 (3.3)	^A^	16.3 (2.1)	^C^	30.4 (1.9)		27.3 (6.8)	^A^
	C	60.3 (4.1)	^B^	59.6 (3.6)	^B^	17.3 (2.1)	^B^	30.3 (1.9)		19.6 (6.7)	^B^
	*P*-value	<0.001		<0.001		<0.001		NS		<0.001	
	SEM	0.12		0.11		0.06		0.05		0.20	

40–50	A	62.3 (4.5)		59.7 (3.4)	^C^	17.4 (2.1)	^A^	30.5 (1.9)	^A^	24.6 (6.1)	^A^
	B	62.2 (4.8)		64.9 (3.9)	^A^	14.3 (2.2)	^B^	29.6 (1.9)	^B^	22.7 (7.3)	^B^
	C	62.1 (6.1)		61.7 (3.5)	^B^	16.8 (2.0)	^A^	30.2 (1.8)	^A^	20.4 (6.4)	^A^
	*P*-value	NS		<0.001		<0.001		<0.001		<0.001	
	SEM	0.14		0.11		0.07		0.05		0.18	

60–70	A	64.2 (5.5)	^A^	60.6 (3.7)	^C^	16.9 (2.2)	^A^	30.5 (1.9)	^A^	27.6 (6.9)	^A^
	B	62.6 (5.0)	^B^	64.7 (4.2)	^A^	14.2 (2.3)	^C^	29.4 (2.0)	^B^	20.7 (7.7)	^B^
	C	61.4 (5.1)	^C^	62.1 (4.2)	^B^	15.4 (2.4)	^B^	29.6 (2.2)	^B^	18.2 (7.5)	^C^
	*P*-value	<0.001		<0.001		<0.001		<0.001		<0.001	
	SEM	0.14		0.12		0.07		0.06		0.22	

Data are expressed as mean and SD (within brackets).

$\Delta E\begin{array}{c} {}*\\ ab\; \end{array}=\;\sqrt{{\left(L\begin{array}{c} {}*\\ 2 \end{array}-L\begin{array}{c} {}*\\ 1 \end{array}\right)}^{2}+{\left(a\begin{array}{c} {}*\\ 2 \end{array}-a\begin{array}{c} {}*\\ 1 \end{array}\right)}^{2}+{\left(b\begin{array}{c} {}*\\ 2 \end{array}-b\begin{array}{c} {}*\\ 1 \end{array}\right)}^{2}\;}$; where “2” and “1” refer respectively to the colorimetric assessments conducted after and before staining. In brief, the higher the ΔE*_ab_ values, the better the staining affinity and thus the eggshell cuticle coverage.

^A^^-^^C^ Means within each interval of age not sharing a common superscript letter are significantly different (*P* < 0.001).

**Table 4:** Effect of hen age (20–30, 40–50, and 60–70 wk) on egg weight, eggshell colorimetric attributes before cuticle staining, and ΔE*_ab_ value in the three hen genotypes (A, B, and C).

Genotype	Age, wks	Egg weight, g		Lightness - L*		Redness - a*		Yellowness - b*		ΔE*_ab_	
A	20–30	60.5 (4.8)	^C^	57.6 (3.3)	^C^	18.4 (2.1)	^A^	30.6 (1.9)		26.1 (6.8)	^B^
	40–50	62.3 (4.5)	^B^	59.7 (3.4)	^B^	17.4 (2.1)	^B^	30.5 (1.9)		24.6 (6.1)	^C^
	60–70	64.2 (5.5)	^A^	60.6 (3.7)	^A^	16.9 (2.2)	^C^	30.5 (1.9)		27.6 (6.9)	^A^
	*P*-value	<0.001		<0.001		<0.001		NS		<0.001	
	SEM	0.13		0.10		0.06		0.05		0.18	

B	20–30	61.4 (4.5)	^B^	63.3 (3.3)	^B^	16.3 (2.1)	^A^	30.4 (1.9)	^A^	27.3 (6.8)	^A^
	40–50	62.2 (4.8)	^B^	64.9 (3.9)	^A^	14.3 (2.2)	^B^	29.6 (1.9)	^B^	22.7 (7.3)	^B^
	60–70	62.6 (5.0)	^A^	64.7 (4.2)	^A^	14.2 (2.3)	^B^	29.4 (2.0)	^B^	20.7 (7.7)	^C^
	*P*-value	<0.001		<0.001		<0.001		<0.001		<0.001	
	SEM	0.13		0.10		0.06		0.05		0.21	

C	20–30	60.3 (4.1)	^B^	59.6 (3.6)	^B^	17.3 (2.1)	^A^	30.3 (1.9)	^A^	19.6 (6.7)	^AB^
	40–50	62.1 (6.1)	^A^	61.7 (3.5)	^A^	16.8 (2.0)	^B^	30.2 (1.8)	^A^	20.4 (6.4)	^A^
	60–70	61.4 (5.1)	^A^	62.1 (4.2)	^A^	15.4 (2.4)	^C^	29.6 (2.2)	^B^	18.2 (7.5)	^B^
	*P*-value	<0.001		<0.001		<0.001		<0.001		<0.001	
	SEM	0.15		0.10		0.06		0.05		0.18	

Data are expressed as mean and SD (within brackets).

$\Delta E\begin{array}{c} {}*\\ ab\; \end{array}=\;\sqrt{{\left(L\begin{array}{c} {}*\\ 2 \end{array}-L\begin{array}{c} {}*\\ 1 \end{array}\right)}^{2}+{\left(a\begin{array}{c} {}*\\ 2 \end{array}-a\begin{array}{c} {}*\\ 1 \end{array}\right)}^{2}+{\left(b\begin{array}{c} {}*\\ 2 \end{array}-b\begin{array}{c} {}*\\ 1 \end{array}\right)}^{2}\;}$; where “2” and “1” refer respectively to the colorimetric assessments conducted after and before staining. In brief, the higher the ΔE*_ab_ values, the better the staining affinity and thus the eggshell cuticle coverage.

^A^^-^^C^Means for each genotype not sharing a common superscript letter are significantly different (*P* < 0.001).

Considering the comparison of genotypes within the same interval of age (Table 3), egg weight (**EW**) showed significant differences only between mean values of groups B and C at 20 to 30 wk (61.4 vs. 60.3 g, respectively; *P* < 0.001), and among all genotypes at 60 to 70 wk (64.2 vs. 62.6 vs. 61.4 g, respectively for A, B, and C; *P* < 0.001).

Eggshell lightness (L*) and redness (a*) values were significantly different among the three genotypes in all the intervals of age, with eggs from A group presenting the lowest values of lightness and the highest of redness (*P* < 0.001). On the other hand, B ones showed the opposite trend being characterized by the highest values of L* and the lowest of b*. Slightly, but still significant, differences were observed for yellowness (b*) values, in particular A and C in respect to B at 40 to 50 wk, and A in respect to B and C at 60 to 70 wk.

Concerning the ΔE*_ab_, eggs from A and B genotypes presented comparable values at 20 to 30 wk, while C ones showed significantly lower cuticle coverage (26.1 and 27.3 vs. 19.6, respectively for A, B, and C; *P* < 0.001). From 40 to 50 wk, the lowest ΔE*_ab_ value was recorded in B group (24.6 vs. 22.7 vs. 20.4, respectively for A, B, and C; *P* < 0.001). In the last interval of age (60–70 wks), the differences among groups were of greater magnitude, with the A group showing the highest value followed by B and C (27.6 vs. 20.7 vs. 18.2, respectively; *P* < 0.001). Overall, our findings revealed a significant effect of the hen genotype on cuticle coverage in each of the age intervals considered in this study. Although available information on this topic is sparse, Bain et al. (2013) hypothesized that genetics might be responsible for an important amount of variation of this trait. Indeed, early findings indicated that cuticle deposition can be affected by the breed (Ball et al., 1975; Sparks, 1994). Similarly, other studies (Simons, 1971; Board and Halls, 1973; Kulshreshtha et al., 2018) reported differences in cuticle properties between brown and white eggs. As the eggshell color is determined by genetic factors, such variations in cuticle quality could be likely associated with the genetic background of the animals. The results obtained in the present study, in which a different capacity in cuticle deposition has been observed among commercial hen genotypes, can have important practical implications especially in countries where egg washing and cleaning cannot be performed for regulatory restrictions. In such countries, consumer's safety relies to a large extent on the role of the cuticle, which is thought, *a priori*, to be present on each egg with no substantial variations among hen strains. Our findings seem questioning this assumption and reinforce the need of including cuticle deposition traits into breeding programs of egg-type chickens to reduce the potential food-borne pathogens transmission associated with egg consumption while enhancing the overall food safety perception of this specific food chain.

As concern the effect of the hen age (Table 4), EW significantly increased in all genotypes passing from 20–30 to 60–70 wk of hen age (+6.1, 1.3, and 1.8%, respectively for genotypes A, B and C; *P* < 0.001). Similarly, the effect of the hen age (20–30 vs. 60–70 wk) was associated with a significant increase in eggshell lightness (L*; +5.2, 2.2 and 4.2%, respectively for A, B, and C; *P* < 0.001) and a reduction of redness (a*; −8.9, 14.8, and 12.3%, respectively; *P* < 0.001). Yellowness (b*) was slightly affected by the hen age in all genotypes. In general, it is widely accepted that egg weight increases with the age of the hens, as also reported in other studies (Tůmová and Gous, 2012; Sirri et al., 2018). As concern the eggshell color profile before staining, the results obtained in the present study corroborate our previous findings in which a significant increase of eggshell lightness (L*) and a reduction of redness (a*) was observed from 30 to 81 wk of hen age (Sirri et al., 2018). Similarly, a limited effect of hen age on eggshell yellowness (b*) was observed also in the present study, confirming the results reported in Sirri et al. (2018).

As for ΔE*_ab_ (Table 4), a significant increase (+5.7%; *P* < 0.001) was observed going from 20–30 to 60–70 wk of hen age in the genotype A. Conversely, a 24.2% reduction was detected in eggs from genotype B over the same period (*P* < 0.001), whereas group C did not evidence a clear trend related to the hen age. Taken together, these results indicate that cuticle deposition in brown eggs is significantly affected by the hen age. However, a significant interaction between genotype and age was observed. Transition from early to late hen age resulted in a significantly different pattern of ΔE*_ab_ changes in each hen strain, as the tested genotypes presented different or even divergent trends for cuticle deposition over the period considered. This outcome could also explain the different results reported in literature as in most of the longitudinal studies investigating the effect of the hen age on cuticle deposition only a single hen genotype has been generally considered. Indeed, if we would have tested only one genotype, we could have concluded that the effect of the hen age on cuticle deposition could be either positive, negative, or not evident according to the results obtained for genotype A, B, and C, respectively. It has been previously reported that white eggs laid by 70-wk-old hens had lower degree of staining and overall cuticle quality than those obtained from younger hens (Rodríguez-Navarro et al., 2013). Similarly, higher amount of cuticle was observed in 44 wk flock eggs in comparison to 64 and 73 wk eggs, which did not differ from each other (Samiullah et al., 2017). Domínguez-Gasca et al. (2017) found that white eggs from 60-wk-old hens have lower amount of cuticle deposited and larger variability in cuticle quality in comparison to those laid by 25-wk-old hens. On the contrary, Bain et al. (2019) reported that cuticle deposition does not decrease with the age in both egg- and meat-type chickens, at least up to 50 wk. Finally, Kulshreshtha et al. (2018) identified a trend for reduced cuticle coverage as hen aged in white eggs but not in brown ones.

**Figure 2:**
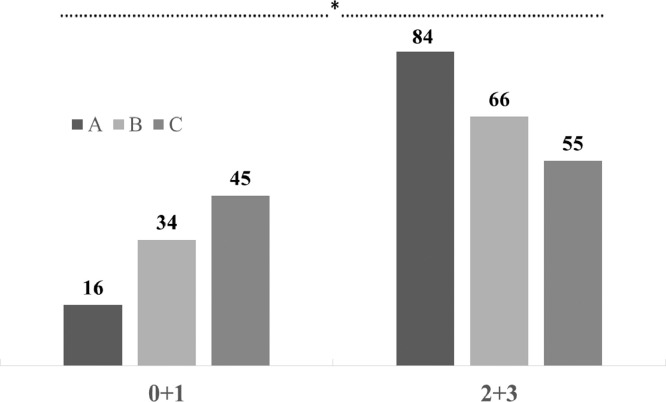
Frequency distribution (%) of the visual score (VS) (0+1 = eggs with impaired cuticle coverage; 2+3 = eggs with total cuticle coverage) according to hen genotypes (A, B and C) (*: *P* < 0.05).

**Figure 3:**
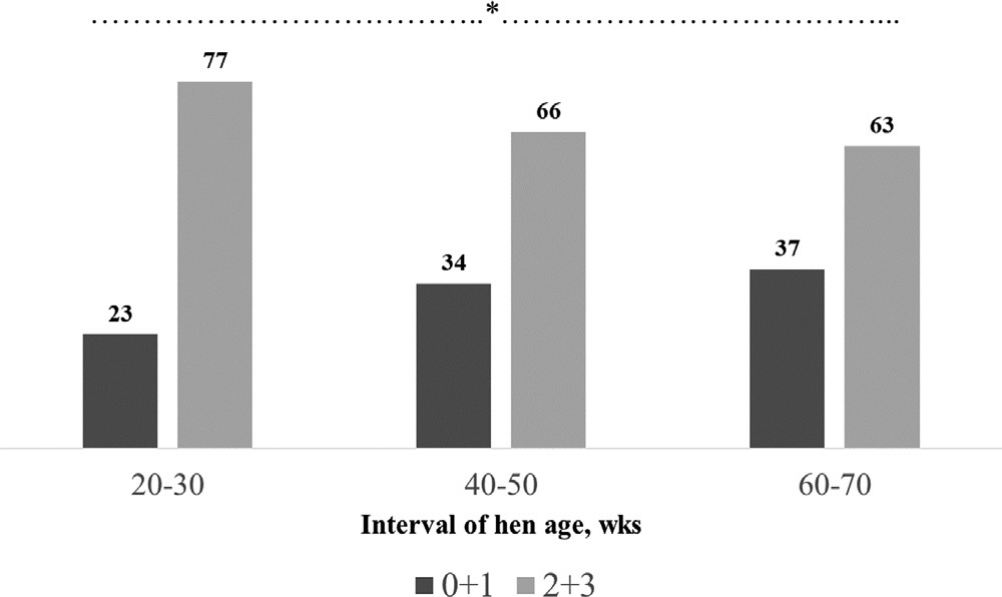
Frequency distribution (%) of the visual score (VS) (0+1 = eggs with impaired cuticle coverage; 2+3 = eggs with total cuticle coverage) according to intervals of age (*: *P* < 0.05).

The frequency distributions of the VS values (% of eggs) according to genotypes and intervals of age are provided in Figure 2. The genotype A presented the highest percentage of eggs (84%) scored 2+3 (total cuticle coverage - low degree and total cuticle coverage - high degree) and the lowest percentage of those (16%) with no/partial cuticle coverage (VS 0+1; *P* < 0.001). On the contrary, group C showed the lowest percentage of eggs (55%) presenting total cuticle coverage - low and high degrees (VS 2+3) and the highest percentage of those (45%) with impaired cuticle coverage (VS 0+1). Intermediate values were recorded for group B (66 and 34%, respectively for VS 2+3 and 0+1). Concerning the effect of the hen age on the VS evaluation (Figure 3), it could be stated that the percentage of eggs showing total cuticle coverage - low and high degrees (VS 2+3) tended to decrease as the hen aged (77, 66, and 63%, respectively for 20–30, 40–50 and 60–70 wks of age; *P* < 0.05), which is consistent with previous findings (Rodríguez-Navarro et al., 2013; Domínguez-Gasca et al., 2017; Samiullah et al., 2017). A positive Spearman's correlation coefficient of 0.68 (*P* < 0.001) was obtained from bivariate correlation analyses conducted between VS and the colorimetric parameter ΔE*_ab_, thereby indicating an optimal correlation between these 2 evaluation methods.

The calculation of Chi-Square, standardized residuals, relative, and absolute contributions of each cell are given in Tables 5 and 6, respectively for hen genotype and interval of age variables. The omnibus Pearson Chi-Square test resulted significant for both genotype and interval of age variables. Different hen genotypes and intervals of age accounted respectively for 11.6 and 2.4% of the variance in the VS. According to Table 5, the standardized residuals in cells for genotypes A and C significantly contributed to the result of the omnibus Chi-Square statistic. Genotypes A and C affected the VS distribution significantly more than expected. In terms of relative contribution (%), largest values were registered for genotype A/VS 3 (27.6%) and “genotype C/VS 3” (26.3%). These cells accounted respectively for 3.2 and 3.1% of shared variance. Apart from cells “age 40 to 50/VS 0_1_3” and “age 60 to 70/VS 2”, hen age affected the cuticle coverage in terms of VS distribution significantly more than expected (Table 6). In terms of relative contribution (%), highest values were identified for cells “age 20 to 30/VS 0” (24.4%) and “age 60 to 70/VS 3” (18.8%). These cells accounted respectively for 0.58 and 0.45% of shared variance.

**Table 5:** Chi-square, standardized residuals, relative, and absolute contribution of visual score (VS) data according to hen genotypes (A, B, and C).

Cell	Observed frequency	Egg (%)	Cell chi-square	Standardized residual	Relative (%) contribution	Absolute (%) contribution
A/0	25	1.8	66.4	−8.2⁎⁎	13.4	1.56
A/1	206	14.4	53.8	−7.3⁎⁎	10.8	1.26
A/2	402	28.2	5.04	−2.2*	1.01	0.12
A/3	795	55.7	137.4	11.7⁎⁎	27.6	3.21

B/0	127	8.9	2.28	1.5	0.46	0.05
B/1	354	24.7	0.39	0.6	0.08	0.01
B/2	431	30.1	0.88	−0.9	0.18	0.02
B/3	520	36.3	0.11	−0.3	0.02	0.01

C/0	180	12.7	44.6	6.7⁎⁎	8.96	1.04
C/1	463	32.7	45.3	6.7⁎⁎	9.11	1.06
C/2	514	36.2	10.2	3.2⁎⁎	2.05	0.24
C/3	261	18.4	130.8	−11.4⁎⁎	26.3	3.06

⁎ Statistical significance at alpha level < 0.05.

⁎⁎ Statistical significance at adjusted-alpha level < 0.01.

**Table 6:** Chi-square, standardized residuals, relative, and absolute contribution of visual score (VS) data for intervals of age (20–30, 40–50, and 60–70 wk).

Intervals of age/VS	Egg (%)	Cell chi-square	Standardized residual	Relative (%) contribution	Absolute (%) contribution
20–30/0	4.1	24.8	−5.0⁎⁎	24.4	0.58
20–30/1	19.6	11.1	−3.3⁎⁎	11.0	0.26
20–30/2	34.6	4.32	2.1*	4.24	0.10
20–30/3	41.7	9.30	3.1⁎⁎	9.14	0.22

40–50/0	8.9	2.57	1.6	2.53	0.06
40–50/1	25.3	1.12	1.1	1.10	0.03
40–50/2	26.9	9.63	−3.1⁎⁎	9.47	0.23
40–50/3	38.9	1.65	1.3	1.62	0.04

60–70/0	10.3	11.7	3.4⁎⁎	11.5	0.27
60–70/1	26.9	5.31	2.3*	5.22	0.12
60–70/2	33.0	1.06	1.0	1.04	0.03
60–70/3	29.8	19.2	−4.4⁎⁎	18.8	0.45

⁎ Statistical significance at alpha level < 0.05.

⁎⁎ Statistical significance at adjusted-alpha level < 0.01.

**Figure 4:**
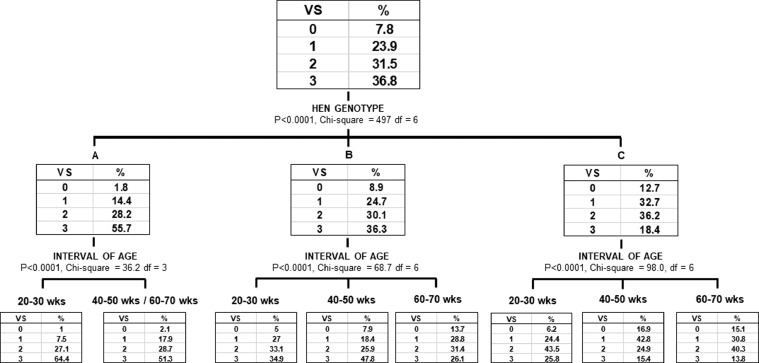
Classification tree (Chi-squared Automatic Interaction Detector) of the explanatory variables hen genotype and interval of age for the occurrence of the response variable visual score (VS).

The classification tree was grown in order to further explore the relationships between the variables “hen genotype” and “interval of age” and the cuticle coverage expressed as VS (Figure 4). The hen genotype, being characterized by the highest chi-square, was found to be the most significant variable affecting the VS. The three subgroups that were identified with the analysis (i.e., A, B and C genotypes) were re-analyzed independently to create further subgroups according to the interval of age variable. Apart from genotype A, where the intervals of ages 40-50 and 60-70 wks were merged in one subgroup, a clear separation can be appreciated for the other 2 genotypes. Overall, these results confirm that the hen genotype had a higher impact on cuticle deposition rather than hen age.

In conclusion, from the present study emerged that the hen genotype and, to a lesser extent, the hen age significantly affect the quality and degree of cuticle coverage as well as the eggshell color profile. Important differences in cuticle deposition capacity were observed among commercial hen genotypes from 20 to 70 wk of age, which can have implications on food safety especially in those countries where egg washing and cleaning cannot be performed for the regulatory framework in force. The significant interaction found between genotype and age on cuticle coverage reveals that hen age impacts differently on this trait in relation to the hen genetic background, which could explain the inconsistent observations reported in literature regarding the effect of the hen age on cuticle deposition. Overall, these findings provide further evidence for considering the importance of cuticle deposition traits into breeding programs of commercial egg-type chickens to reduce the risk of foodborne pathogen transmission associated with egg consumption while enhancing the overall food safety perception of this supply chain.
